# Intensive care drug therapy and its potential adverse effects on blood pressure and heart rate in critically ill children

**DOI:** 10.1007/s12519-023-00683-0

**Published:** 2023-02-28

**Authors:** Lisa Marie Kiesel, Astrid Bertsche, Wieland Kiess, Manuela Siekmeyer, Thilo Bertsche, Martina Patrizia Neininger

**Affiliations:** 1 Drug Safety Center and Clinical Pharmacy, Institute of Pharmacy, Medical Faculty, Leipzig University, Bruederstr. 32, 04103 Leipzig, Germany; 2 Division of Neuropediatrics, University Hospital for Children and Adolescents, Fleischmannstr. 8, 17475 Greifswald, Germany; 3 Department of Women and Child Health, Hospital for Children and Adolescents and Center for Pediatric Research, University Hospital of Leipzig, Liebigstr. 20a, 04103 Leipzig, Germany

**Keywords:** Adverse drug reaction, Drug therapy, Hemodynamic monitoring, Multifactorial causality, Pediatric intensive care unit

## Abstract

**Background:**

Owing to complex treatment, critically ill children may experience alterations in their vital parameters. We investigated whether such hemodynamic alterations were temporally and causally related to drug therapy.

**Methods:**

In a university pediatric intensive care unit, we retrospectively analyzed hemodynamic alterations defined as values exceeding the limits set for heart rate (HR) and blood pressure (BP). For causality assessment, we used the World Health Organization–Uppsala Monitoring Center (WHO–UMC) system, which categorizes the probability of causality as “certain,” “probable,” “possible,” and “unlikely.”

**Results:**

Of 315 analyzed patients with 43,200 drug prescriptions, 59.7% experienced at least one hemodynamic alteration; 39.0% were affected by increased HR, 19.0% by decreased HR, 18.1% by increased BP, and 16.2% by decreased BP. According to drug information databases, 83.9% of administered drugs potentially lead to hemodynamic alterations. Overall, 88.3% of the observed hemodynamic alterations had a temporal relation to the administration of drugs; in 80.2%, more than one drug was involved. Based on the WHO–UMC system, a drug was rated as a “probable” causing factor for only 1.4% of hemodynamic alterations. For the remaining alterations, the probability ratings were lower because of multiple potential causes, e.g., several drugs.

**Conclusions:**

Critically ill children were frequently affected by hemodynamic alterations. The administration of drugs with potentially adverse effects on hemodynamic parameters is often temporally related to hemodynamic alterations. Hemodynamic alterations are often multifactorial, e.g., due to administering multiple drugs in rapid succession; thus, the influence of individual drugs cannot easily be captured with the WHO–UMC system.

**Supplementary Information:**

The online version contains supplementary material available at 10.1007/s12519-023-00683-0.

## Introduction

Patients treated in a pediatric intensive care unit (PICU) often have a complex medical history, a severe underlying disease, or are in an acute life-threatening condition [1, 2]. Therefore, they usually need comprehensive drug therapy, which may result in adverse drug reactions (ADRs), such as unexpected alterations in vital parameters. Previous studies on the prevalence of ADR in critically ill children have reported ADR rates ranging from 4.9 to 20.5 ADR per 100 patient days [3, 4]. In addition, alterations in hemodynamic parameters, such as increased heart rate (HR) or increased blood pressure (BP), frequently occur in critically ill children [5, 6]. Hemodynamic alterations can lead to complications, such as inadequate tissue perfusion, and thus to patient harm [7]. Therefore, monitoring hemodynamic parameters is one of the key components in the clinical assessment of critically ill patients [8]. In numerous previous studies dealing with ADRs, hemodynamic alterations have not yet been taken into account comprehensively [4, 9–11]. If hemodynamic alterations were detected, they represented only a minor proportion of the overall number of ADRs. A separate assessment of hemodynamic alterations, e.g., regarding drugs that may have led to this ADR, was not performed [4, 9–11]. Therefore, there is a lack of knowledge about the frequency of those ADRs and about the drugs frequently involved in hemodynamic alterations.

In this study, we analyzed data from children treated in a PICU to identify hemodynamic alterations, defined as deviations from the standard ranges of HR and BP adjusted by the treating physicians according to the patient’s health condition. The objective of this study was to investigate the impact of drugs with potential adverse reactions on hemodynamic parameters in pediatric intensive care patients. Therefore, we analyzed the administration of such drugs in pediatric intensive care and aimed to evaluate whether they were temporally related to hemodynamic alterations. Furthermore, we investigated whether causal relations between administered drugs and subsequent alterations can be established using the WHO–UMC (World Health Organization—Uppsala Monitoring Center) system. The WHO–UMC system is widely used by clinicians [12, 13]. However, it is unclear whether it is appropriate for identifying causal relations between drug administration and subsequent hemodynamic alterations in pediatric intensive care. Thus, we aimed to determine whether the WHO–UMC system is helpful in identifying ADRs in pediatric intensive care.

## Methods

### Setting and study design

We performed a retrospective study in the PICU of a university hospital. We evaluated the data of patients hospitalized from April 1, 2018, to March 30, 2019. An electronic patient data management system was available in the unit. Data from all patients with a stay of at least 48 h in the PICU were included. As the university hospital has a separate pediatric oncology unit, we excluded patients who were receiving chemotherapy, as they were only transferred to the PICU for a short time if their health condition deteriorated dramatically. Substances for volume replacement therapy, e.g., Ringer’s acetate solution or isotonic saline solution, and total parenteral nutrition were also not included in the evaluation.

### Ethics

The study was approved by the local ethics committee. As this was a retrospective study and data were collected from patient records without any influence on patients’ treatment, the ethics committee waived informed consent.

### Identification of hemodynamic alterations

We aimed to detect hemodynamic alterations based on the standard ranges of HR and BP for children and adolescents derived from the literature [14–21] (Supplemental Table 1). For some patients, the attending physicians adjusted the limits according to the patient’s health condition. An adjustment occurred mainly in patients after major surgery or in patients with respiratory or gastrointestinal infections. To detect clinically relevant hemodynamic alterations for each patient, a clinical pharmacist assessed HR and BP based on bedside nurses’ and physicians’ daily documentation of HR and BP. For these documented hemodynamic alterations, we also reviewed the recorded values by the automated intensive care monitoring devices to obtain the exact values that exceeded the thresholds. We distinguished between increased or decreased HR and increased or decreased BP.

### Identification of drugs potentially leading to hemodynamic alterations

We used standard drug databases to investigate whether each administered drug could lead to a hemodynamic alteration. We reviewed three drug information databases: UpToDate (provided by Wolters Kluwer, Riverwoods, Illinois, USA), drugs.com (provided by Drugsite Trust, Auckland, New Zealand), and ABDA-Database (a standard drug information system in German community pharmacies and hospitals provided by the Federal Union of German Associations of Pharmacists, Eschborn, Germany). We used more than one database because previous studies reported differences in ADR specifications and frequency classifications between various drug information databases [22–24]. A drug was classified as potentially leading to a hemodynamic alteration such as increased HR if the particular alteration was listed in at least one of the databases for the respective drug. Adapted for the databases, the following frequency classifications were assigned to quantify how often a drug could potentially lead to a hemodynamic alteration: “common” (from 1% up), “uncommon” (0.1% to 1%) and “rare” (below 0.1%). If the frequencies differed among the databases, the highest frequency classification reported was used for further analysis to ensure a standardized evaluation process and not to prefer one of the three databases.

### Temporal relation between administered drugs and hemodynamic alterations

In assessing the temporal relations between administered drugs and subsequent hemodynamic alterations, we analyzed how many drugs that could lead to the specific hemodynamic alteration were administered to a patient within 24 hours before the alteration occurred. Thus, we identified drugs that might be a relevant causative factor for alterations due to their administration prior to hemodynamic alterations. Furthermore, for each drug assigned to the frequency class of “common” for the potential to lead to a hemodynamic alteration, we analyzed the total number of patients who received the drug and how many of them experienced the specific hemodynamic alteration within 24 hours after the administration. We used a standardized 24-hour interval to account for drugs with either short- or long-acting mechanisms resulting in immediate or delayed hemodynamic alterations, respectively.

### Causal relations between administered drugs and hemodynamic alterations

We evaluated the causal relations between administered drugs and hemodynamic alterations to investigate whether the WHO–UMC system is appropriate for the pediatric intensive care setting. We only considered drugs potentially leading to the related hemodynamic alteration and administered within 24 hours before the alteration occurred. Using the WHO–UMC assessment, we also took into account several different factors, such as the patient’s condition, that could have influenced the occurrence of the hemodynamic alteration. In this evaluation, every drug potentially leading to a particular hemodynamic alteration was considered regardless of its frequency classification from the databases. The WHO–UMC system considers the chronological relationship, other possible causes for the alteration, and reaction to re-challenge or discontinuation of the drug. Additionally, the method of drug administration as well as the dosage interval and duration of drug administration were taken into account in the causality assessment. The assessment distinguishes the causality categories: “certain,” “probable,” “possible,” and “unlikely” [13].

## Results

### Characteristics of patients and administered drugs

We assessed data from 315 patients who had a total of 3788 hospital days in the PICU and 43,200 drug prescriptions (Table 1). With respect to the standard ranges for HR and BP presented in Supplemental Table 1, the lower HR limits complied with the standard ranges in 87.3% (275/315) of patients, and the upper HR limits complied in 79.7% (251/315) of patients. Regarding BP, the lower BP limits were within the standard range in 85.7% (270/315) of patients, and the upper BP limits were within the standard range in 100% (315/315) of patients (Supplemental Table 2). During the study period, 255 different drugs were administered to the patients. Table 2 shows that patients most frequently received analgesics (280/315, 88.9%), followed by antacids and acid reducers (268/315, 85.1%), and supplementation of minerals, vitamins, or miscellaneous ingredients such as pancreatic enzymes (257/315, 81.6%).

**Table 1:** Characteristics of the study population

Characteristics	Value
Number of patients (m/f), *n*	315 (183/132)
Median age (Q25/Q75; min/max), y	3.73 (0.82/11.32; 0.00/22.79)
Median length of stay in hospital (Q25/Q75; min/max), d	13 (8.5/22; 3/394)
Median length of stay on PICU (Q25/Q75; min/max), d	8 (4/14; 3/99)
Death, *n*/*N* (%)	6/315 (1.9)
Median number of drugs per patient per day (Q25/Q75; min/max), *n*	10 (7/15; 1/34)
Diagnosis according ICD-10 leading to admission*, n*/*N* (%)	
Diseases of the respiratory system	60/315 (19.1)
Diseases of the nervous system	31/315 (9.8)
Other congenital malformations of the digestive system	25/315 (7.9)
Neoplasms (without chemotherapy, surgical treatment only e.g., resection of the tumor)	25/315 (7.9)
Diseases of the digestive system	24/315 (7.6)
Diseases of the musculoskeletal system and connective tissue	22/315 (7.0)
Congenital malformations and deformations of the musculoskeletal system	18/315 (5.7)
Injury, poisoning and certain other consequences of external causes	18/315 (5.7)
Certain infectious and parasitic diseases	15/315 (4.8)
Endocrine, nutritional and metabolic diseases	12/315 (3.8)
Diseases of the urogenital system	9/315 (2.9)
Certain conditions originating in the perinatal period	8/315 (2.5)
Congenital malformations of the nervous system	7/315 (2.2)
Symptoms and abnormal clinical and laboratory findings not elsewhere classified	7/315 (2.2)
Chromosomal abnormalities, not elsewhere classified	5/315 (1.6)
Congenital malformations of the circulatory system	5/315 (1.6)
Cleft lip and cleft palate	4/315 (1.3)
Miscellaneous	20/315 (6.4)

*ICD-10* International classification of diseases 10th revision. *m* male, *f* female, *min* minimum, *max* maximum, *Q25* 25% quantile, *Q75* 75% quantile

**Table 2:** Top 20 administered drug classes according to the ATC (anatomical therapeutic chemical classification) system

Drug class	Frequency *n*	Frequency % (/315 patients)
Analgesics	280	88.9
Antacids and acid reducers	268	85.1
Mineral, vitamins, and miscellaneous supplementation	257	81.6
Anti-infective	223	70.8
Sedatives	195	61.9
Antiemetics	147	46.7
Diuretics	140	44.4
Laxatives	126	40.0
Bronchodilators	125	39.7
Anticoagulants	112	35.6
Anticonvulsants	109	34.6
Glucocorticoids	103	32.7
Decongestants	96	30.5
Mucolytics	70	22.2
Carminatives	33	10.5
Stimulators of cardiovascular system	28	8.9
Thyroid hormones	27	8.6
Antihypertensives	19	6.0
Respiratory stimulants	17	5.4
Muscle relaxants	16	5.1

### Observed hemodynamic alterations

**Fig.1:**
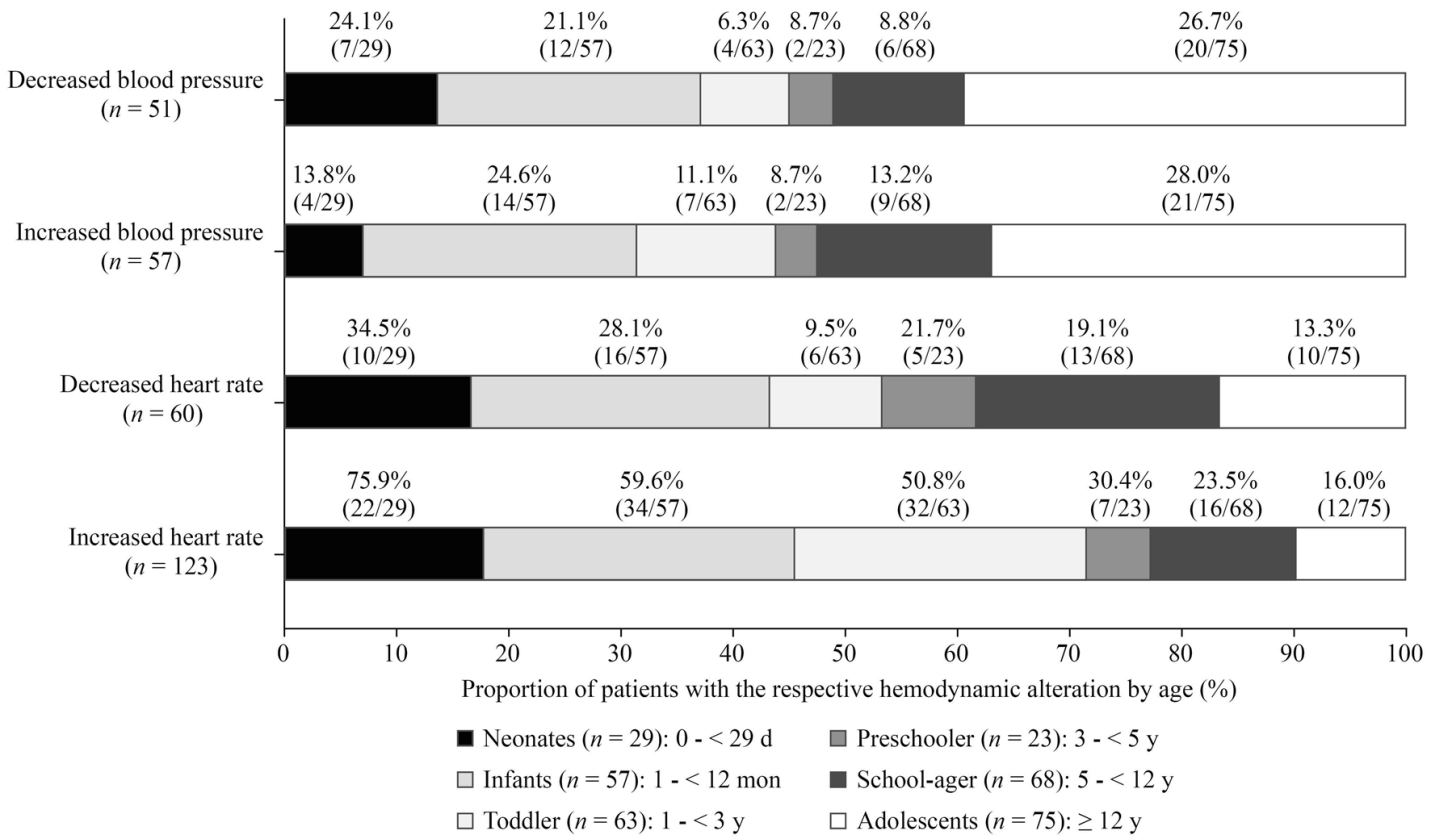
Proportion of patients with the respective hemodynamic alteration by age. The age distribution of patients affected by the respective hemodynamic alteration varied between alterations. In addition, the proportion of patients affected in the respective age groups is shown.

At least one hemodynamic alteration occurred in 188/315 (59.7%) patients during their PICU stay. In total, 1183 hemodynamic alterations were detected. We calculated a rate of 31.2 hemodynamic alterations per 100 patient days (1183 hemodynamic alterations/3788 patient days). Patients were most frequently affected by increased HR [123/315 (39.0%) patients, 597/1183 (50.5%) alterations], followed by decreased HR [60/315 (19.0%) patients, 166/1183 (14.0%) alterations], increased BP [57/315 (18.1%) patients, 273/1183 (23.1%) alterations], and decreased BP [51/315 (16.2%) patients, 147/1183 (12.4%) alterations]. The deviations of the measured values from the standard ranges are shown in Table 3. Considering age, the patients with increased HR were the youngest (median age: 1.2 years; Q25/Q75: 0.2/4.1; min/max: 0.0/20.2), followed by patients with decreased HR (median: 2.1 years; Q25/Q75: 0.3/9.4; min/max: 0.0/17.1), patients with increased BP (median: 6.2 years; Q25/Q75: 0.5/14.2; min/max: 0.0/20.2), and patients with decreased BP (median: 7.2 years; Q25/Q75: 0.4/14.9; min/max: 0.0/20.2; Fig. 1).

**Table 3:** Deviations of measured hemodynamic parameters from the standard ranges of blood pressure and heart rate

Hemodynamic alteration	Median deviation (%)	Q25 (%)	Q75 (%)	Min (%)^a^	Max (%)
Increased heart rate	5.6	2.8	11.1	0.0	27.8
Decreased heart rate	14.6	10.0	25.0	0.0	62.5
Increased blood pressure–systolic	18.1	6.6	28.0	2.4	53.9
Increased blood pressure–diastolic	16.9	8.9	29.9	1.3	42.9
Decreased blood pressure–systolic	18.8	7.1	30.6	0.0	100.0
Decreased blood pressure–diastolic	32.4	18.9	45.5	0.0	56.5

^a^If a value was measured being equal to the outer limit, this was also rated as hemodynamic alteration

*min* minimum, *max* maximum, *Q25* 25% quantile, *Q75* 75% quantile

### Drugs with the potential to lead to hemodynamic alterations

Of the different drugs administered to the patients during the study period, 214/255 (83.9%) could lead to at least one hemodynamic alteration, according to the databases. More specifically, 123/255 (48.2%) drugs were associated with an increased HR. For 35/255 (13.7%) drugs, the frequency of increased HR was classified as “common” according to the databases. Decreased HR was associated with 86/255 (33.7%) drugs [“common”: 16/255 (6.3%)], increased BP with 97/255 (38.0%) drugs [“common”: 36/255 (14.1%)], and decreased BP with 145/255 (56.9%) drugs [“common”: 56/255 (22.0%)].

During the stay in the PICU, every patient [315/315 (100.0%)] was administered at least one drug that could lead to an increased HR. For decreased HR and increased BP, almost all patients [each 305/315 (96.8%)] were given at least one drug with the potential to lead to the respective hemodynamic alteration. For decreased BP, 314/315 (99.7%) patients received at least one drug potentially associated with this alteration.

### Temporal relation between drug administration and observed hemodynamic alteration

In 1045/1183 (88.3%) hemodynamic alterations, we found a temporal relationship between drug administration and adverse reactions (Table 4). Table 4 also shows that 949/1183 (80.2%) hemodynamic alterations were temporally related to more than one drug. A total of 5963 administered drugs were temporally associated with hemodynamic alterations.

**Table 4:** Temporal relations between drug administration and subsequent hemodynamic alterations within 24 h after administration

Hemodynamic alteration	Total number of hemodynamic alterations *n/N* (%)	Number of hemodynamic alterations temporally related to drug therapy *n/N* (%)	Number of hemodynamic alterations temporally related to more than one drug *n/N* (%)	Median number of drugs with the potential to lead to the hemodynamic alteration that patients received within 24 h before the alteration occurred (Q25/Q75; min/max) *n*
Increased heart rate	597/597 (100.0)	550/597 (92.1)	494/597 (82.7)	5 (2/8; 0/17)
Decreased heart rate	166/166 (100.0)	105/166 (63.3)	91/166 (54.8)	2 (0/4; 0/10)
Increased blood pressure	273/273 (100.0)	249/273 (91.2)	225/273 (82.4)	3 (2/5; 0/11)
Decreased blood pressure	147/147 (100.0)	141/147 (95.9)	139/147 (94.6)	8 (5/10; 0/19)

**Fig. 2:**
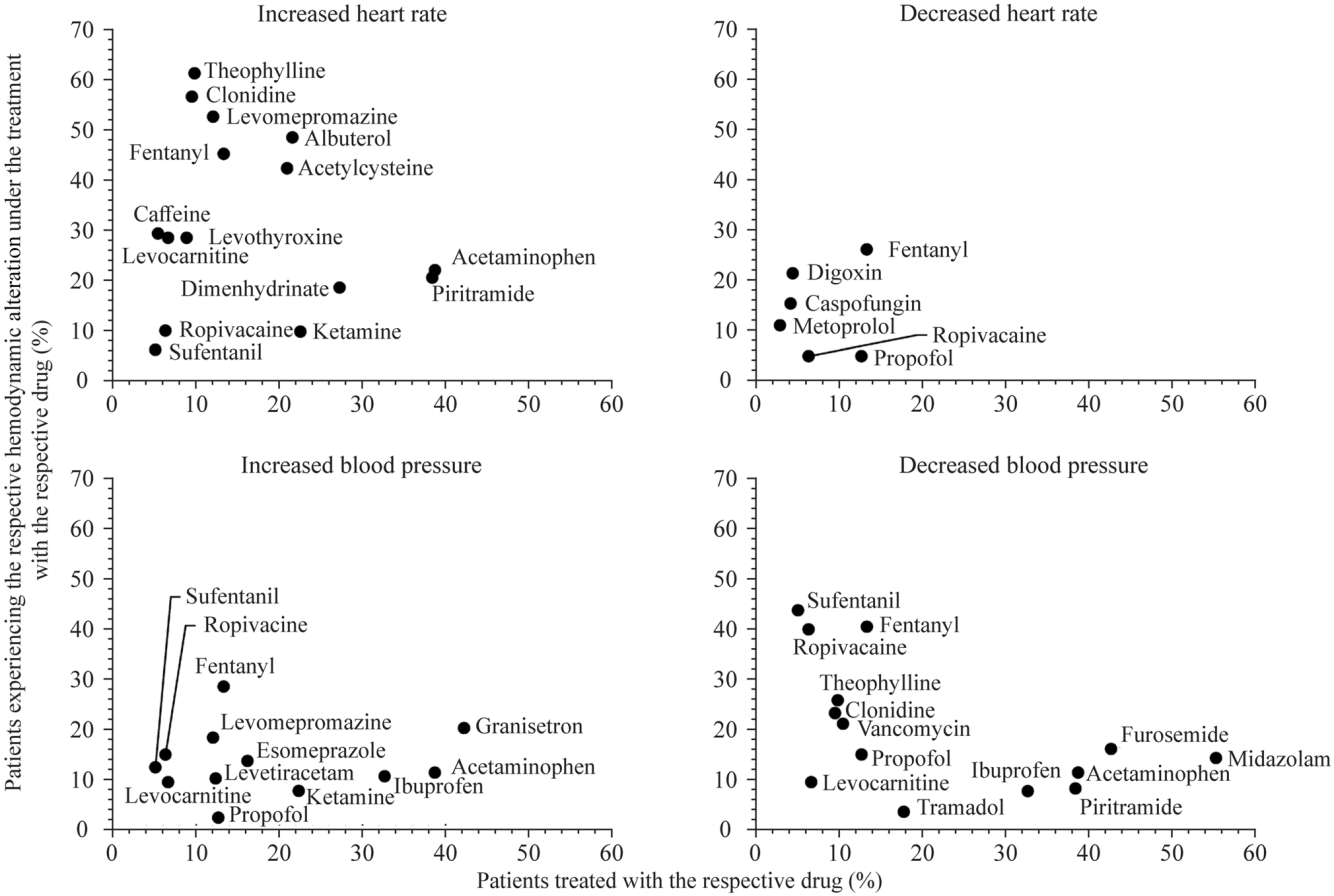
Specific drugs temporally associated with hemodynamic alterations. For each observed hemodynamic alteration, the number of patients who received a drug is plotted against the number of patients in whom the respective alteration occurred during therapy with the specific drug; only drugs whose reported frequency was classified as “common” according to the database search were considered; drugs administered in at least 5% of patients are shown.

Considering only drugs with the frequency class of “common” reported in the databases, we found 15 specific drugs that were temporally related to increased HR (Fig. 2). Decreased HR was temporally associated with 6 specific drugs, increased BP with 12 specific drugs, and decreased BP with and decreased BP with 14 specific drugs. Although all these specific drugs were categorized into the same frequency class of “common” according to the databases, Fig. 2 shows variability in the number of patients affected by the respective hemodynamic alteration. For example, acetaminophen was administered to 122/315 (38.7%) patients, of whom 27/122 (22.1%) were subsequently affected by increased HR. Levomepromazine was administered to 38/315 (12.0%) patients, of whom 20/38 (52.6%) subsequently had an increased HR.

### Causal relation of drug therapy to observed hemodynamic alterations

To assess the causal relationship between drug administration and a subsequent hemodynamic alteration, we used the WHO–UMC system to analyze each of the 5963 drug administrations for which a temporal relation to an alteration was found (Supplemental Table 3). In 1020/5963 (17.1%) of the administered drugs, a causal connection was assessed as “unlikely.” In 4924/5963 (82.6%) drug administrations, the causal relations between administered drugs and hemodynamic alteration were rated as “possible,” according to WHO–UMC. The highest causality rating in the study was “probable” and affected 19 drugs (Table 5). No causal relationship between drug administration and a hemodynamic alteration was classified as “certain.” In detail, we assessed a drug as a “probable” causative factor for 7/597 (1.2%) events of increased HR, for 2/166 (1.2%) events of decreased HR, for 2/273 (0.7%) events of increased BP and for 4/147 (2.7%) events of decreased BP. For 2/147 (1.4%) events of decreased BP, a continuous epidural infusion of ropivacaine with sufentanil was rated as a “probable” causative factor because a separate rating of each drug was not feasible due to the mixed infusion. Thus, we found a “probable” causal relation for 17/1183 (1.4%) hemodynamic alterations.

**Table 5:** Drugs causing the respective hemodynamic alteration, with a rating of “probable” according to the WHO–UMC system

Hemodynamic alterations	Frequency classification of drugs according to databases	
	Common	Rare
Increased heart rate	Norepinephrine	Theophylline
	Albuterol; inhaled	Reproterol^a^
	Dopamine	Epinephrine; inhaled
Decreased heart rate	Metoprolol; oral	Not applicable
	Fentanyl	
Increased blood pressure	Not applicable	Methylprednisolone; oral
		Norepinephrine
Decreased blood pressure	Clonidine	Levomepromazine
	Sufentanil^a^	
	Ropivacaine^a^	
	Midazolam	
	Propofol	

Unless otherwise stated, drugs were administered by infusion or injection. Drugs with a frequency classification of “uncommon” were not identified as “probable” cause

^a^These drugs were assessed twice as “probable” causative factor for the respective hemodynamic alteration. All other drugs were assessed only once as “probable” causative factor

## Discussion

This study shows that hemodynamic alterations frequently occur in the pediatric intensive care setting. According to the databases, many drugs administered during a stay in a PICU have the potential to lead to alterations in HR or BP. We often observed those drugs being administered in rapid succession before a hemodynamic alteration occurred. Consequently, administering drugs with the potential to lead to hemodynamic alterations in rapid succession may trigger or exacerbate the actual occurrence of alterations in HR and BP in critically ill children. However, the WHO–UMC system, a frequently used method to identify ADRs in routine care, rarely identified a strong causal relation between the administered drug and a hemodynamic alteration. One of the reasons for the failure to identify strong causal relations between a single drug and an ADR is the design of the WHO–UMC system. If there are several possible causes, this leads to a strong reduction in the probability assessment of a single drug. However, it is common in pediatric intensive care that several causes are possible. Therefore, a low causality rating should not result in the general conclusion that an adverse reaction is not drug related; this oversight could lead to therapeutic misjudgment. In its current design, the use of the WHO–UMC system is limited in the PICU setting because of its monofactorial approach to causality assessment.

### Hemodynamic alterations are common symptoms in the PICU

In the present study, two-thirds of all patients were affected by at least one hemodynamic alteration during their PICU stay. Most patients were affected by increased HR, followed by decreased HR, increased BP and decreased BP. Previous studies stated that alterations of the hemodynamic balance are common in the pediatric intensive care setting but did not report precise numbers of affected children [5, 6]. Du et al. identified drug therapy as a factor associated with the occurrence of hemodynamic alterations. They suggested close monitoring of patients with risk factors for ADRs to enhance patient safety [9]. Unquestionably, not every hemodynamic alteration will lead to a clinically relevant event. However, close monitoring, as was done in our study, may allow early detection of alterations so that timely adjustment of intensive care treatment can prevent alterations leading to clinically relevant adverse reactions. In this context, children younger than 1 year seem to be more frequently affected by alterations of HR and should be monitored accordingly. In adolescents, however, attention should be given to alterations of BP, as we have observed these more frequently in this age group.

### Drug therapy is a factor frequently associated with hemodynamic alterations

In our study, many drugs that could trigger or exacerbate hemodynamic alterations were administered to patients. According to the databases, 57% of the administered drugs could potentially lead to decreased BP, 48% to increased HR, 38% to increased BP and 34% to decreased HR. Nearly every tenth drug was described in at least one database as “commonly” involved in increased HR, increased BP or decreased HR, and every fifth drug was described as “commonly” leading to decreased BP. In our study, every patient received at least one drug during intensive care that could lead to a hemodynamic alteration. Accordingly, we identified numerous temporal relations between administered drugs and subsequent alterations.

In most cases, several drugs with the potential to cause the related hemodynamic alteration were administered in rapid succession before the alterations occurred. For example, before an increased HR occurred, which affected almost half of the patients, a median number of five drugs had been administered that could lead to increased HR. However, these drugs cannot always be substituted. For this reason, physicians should pay particular attention when several drugs are administered that potentially lead to the same alteration. As we assumed that better knowledge of drugs that are frequently temporally associated with hemodynamic alterations could enable more targeted monitoring, we aimed to identify the most relevant. We identified 15 specific drugs, e.g., albuterol, fentanyl and acetaminophen, which were often administered before an increased HR occurred. These drugs are among the most frequently administered in pediatric intensive care [25, 26]. Even though all of those drugs had the same frequency classification of “common” according to the databases, the drugs varied in their actual frequency of causing increased HR. We also observed this phenomenon for the other three hemodynamic alterations. To prevent harm, physicians should be aware of drugs frequently associated with altered vital parameters, such as HR and BP [27, 28]. Since many drugs have the potential to lead to hemodynamic alterations, it is often difficult for clinicians to find a therapeutic alternative. However, alternative drugs should be substituted whenever possible, especially if the patient receives several drugs that may lead to a hemodynamic alteration or if the patient already suffers from increased HR or BP [28, 29].

### Challenges in the assessment of causal relations between drug therapy and hemodynamic alterations

Using the WHO–UMC system for causality assessment, only 19 of almost 6000 analyzed potential causal relations between administered drugs and subsequent hemodynamic alterations were rated as “probable.” A higher rating was often not achieved because hemodynamic alterations are mostly multifactorial events due to the multidimensional treatment and complex health status of critically ill children [30]. Increased HR, for example, could also result from pain, fever, stress due to inadequate sedation, major surgery or a combination of multiple drugs [6, 31]. If more than one factor is considered potentially causative for an ADR, the probability ratings of conventional methods for causality assessment, such as the WHO–UMC system, are substantially lowered because of the limited ability of these methods to assess multiple causes simultaneously [32–34]. Consequently, the causal relation between drug therapy and hemodynamic alteration was most often rated as “possible”, as also desribed in the literature [35, 36].

In contrast, probabilistic or Bayesian approaches can evaluate multiple causes simultaneously [34]. However, these methods require considerable time, complex calculations, and technical and human resources and are not widely used in routine care [34]. Based on our study results, we conclude that the WHO–UMC system in its current version is of limited use for assessing causal relations in pediatric intensive care, as also observed in previous studies in other pediatric settings [35, 37]. When clinicians use the WHO–UMC system, they need to be aware that it can easily underestimate the actual causal contribution of a drug to a hemodynamic alteration. In our study, hemodynamic alterations often followed the administration of several drugs that can cause these alterations. However, the administration of such drugs in rapid succession lowered the rating of each drug in the per-event causality assessment. This makes it challenging for physicians to assess which specific drug is the cause of the adverse reaction. Due to the limited performance of the WHO–UMC system in the PICU setting, false negative assessments, in particular, may occur. This means that potentially harmful ADRs are not recognized as such, and consequently, drug therapy is not appropriately adjusted. Therefore, the WHO–UMC system should be further developed to make it more appropriate for pediatric intensive care. For example, the assessment could be adapted so that the rating is not lowered when multiple drugs are involved.

### Limitations

The study has some limitations that should be considered when interpreting the results. We chose a 24-hour interval to analyze the temporal relationship between drug administration and subsequent hemodynamic alterations. We know that many hemodynamic alterations may occur in a much shorter period after drug administration. However, we endeavored to enable a standardized assessment and to take into account both short-acting and long-acting effects. We also did not investigate whether the dosage of a particular drug influenced the occurrence or severity of the hemodynamic alterations. Additionally, when assessing the temporal and causal relations between drug therapy and hemodynamic alterations, we did not differ between various grades of severity of the observed hemodynamic alteration. Since even small changes can lead to harm in the vulnerable group of critically ill children, we aimed to detect the influence of drug therapy on any alterations in HR and BP.

### Conclusion

Hemodynamic alterations were seen in two-thirds of the critically ill pediatric patients in our study. More than 80% of drugs administered in the PICU could lead to hemodynamic alterations. These drugs may trigger or exacerbate hemodynamic alterations, especially if several are administered in rapid succession. We found a temporal relationship between drug administration and subsequent alterations for almost 90% of observed hemodynamic alterations. In contrast, we found only a few strong causal relations using the WHO–UMC system. The WHO–UMC system was designed to associate causality with a single drug, but multiple drugs are routinely used in pediatric intensive care. Accordingly, treating physicians should bear in mind that a drug with a low rating may nevertheless be involved in a hemodynamic alteration. Finally, causality assessment methods are needed that more comprehensively reflect the multifactorial nature of adverse effects in pediatric intensive care.

## Electronic supplementary material

Below is the link to the electronic supplementary material.

Supplementary material 1 (PDF 87 KB)

Supplementary file2 (MP4 74337 KB)

## Data Availability

The datasets generated during and/or analyzed during the current study are available from the corresponding author on reasonable equatorial nature of adverse reactions in pediatric intensive care.
