# Supplementary material for: Intensive care drug therapy and its potential adverse effects on blood pressure and heart rate in critically ill children
Source: World J Pediatr. 2023 Feb 28;19(9):902–11. doi: 10.1007/s12519-023-00683-0 (PMC10423157; doi:10.1007/s12519-023-00683-0)
Supplement: Supplementary file 1 — Supplementary material 1 (PDF 87 KB) [file 12519_2023_683_MOESM1_ESM.pdf]

Supplemental Table 1. Standard ranges of heart rate and blood pressure according to age derived from the literature and used in the pediatric intensive care unit

Age	Heart Rate, bpm	Blood Pressure, mmHg	
		Systolic	Diastolic
<12 months	80 - 180	72 - 104	37 - 56
1 - <2 years	80 - 180	80 - 113	46 - 79
2 - <3 years	60 - 150	80 - 113	46 - 79
3 - <4 years	60 - 140	80 - 113	46 - 79
4 - <5 years	60 - 140	80 - 115	47 - 79
5 - <6 years	60 - 140	80 - 115	47 - 79
6 - <7 years	60 - 140	80 - 115	47 - 79
7 - <8 years	60 - 140	83 - 122	52 - 83
8 - <9 years	60 - 140	83 - 122	52 - 83
9 - <10 years	60 - 140	83 - 122	52 - 83
10 - <11 years	50 - 120	83 - 122	52 - 83
11 - <12 years	50 - 120	95 - 136	58 - 88
12 - <13 years	50 - 120	95 - 136	58 - 88
13 - <14 years	50 - 110	100 - 127	55 - 77
≥14 years	50 - 110	100 - 127	55 - 77

bpm: beats per minute; mmHg: millimeter of mercury

Standard ranges vary in the literature. Standard ranges were derived from [14-21].

Supplemental Table 2. Physicians' individual patient adjustments of the standard ranges

	Frequency of affected patients, *n/N*	Frequency of affected patients, %
**Heart rate: Adjustment of the lower limit of the standard range of heart rate**		
0 - 10 bpm	25/315	7.9
11 - 20 bpm	15/315	4.8
**Heart rate: Adjustment of the upper limit of the standard range of heart rate**		
0 - 10 bpm	23/315	7.3
11 - 20 bpm	22/315	7.0
21 - 30 bpm	19/315	6.0
**Blood pressure: Adjustment of the lower limit of the standard range of systolic blood pressure**		
10 - 20 mmHg	45/315	14.3
**Blood pressure: Adjustment of the lower limit of the standard range of diastolic blood pressure**		
10 - 20 mmHg	45/315	14.3

bpm: beats per minute; mmHg: millimeter of mercury

There was no adjustment of the upper limit of the standard ranges of systolic or diastolic blood pressure.

Supplemental Table 3. Assessment of the causal relationship between administered drugs with the potential to lead to hemodynamic alterations and actually occurring hemodynamic alterations with respect to the different frequency classifications according to the database search

Frequency classification of drugs according to databases		WHO-UMC causality criteria	Increased heart rate *n/N* (%)	Decreased heart rate *n/N* (%)	Increased blood pressure *n/N* (%)	Decreased blood pressure *n/N* (%)
	Common	Certain	0/3419 (0.0)	0/429 (0.0)	0/930 (0.0)	0/1185 (0.0)
		Probable	3/3419 (0.1)	2/429 (0.5)	0/930 (0.0)	7/1185 (0.6)
		Possible	869/3419 (25.4)	31/429 (7.2)	407/930 (43.8)	413/1185 (34.8)
		Unlikely	182/3419 (5.3)	8/429 (1.9)	48/930 (5.2)	70/1185 (5.9)
	Uncommon	Certain	0/3419 (0.0)	0/429 (0.0)	0/930 (0.0)	0/1185 (0.0)
		Probable	0/3419 (0.0)	0/429 (0.0)	0/930 (0.0)	0/1185 (0.0)
		Possible	571/3419 (16.7)	31/429 (7.2)	111/930 (11.9)	268/1185 (22.6)
		Unlikely	70/3419 (2.1)	13/429 (3.0)	10/930 (1.1)	33/1185 (2.8)
	Rare	Certain	0/3419 (0.0)	0/429 (0.0)	0/930 (0.0)	0/1185 (0.0)
		Probable	4/3419 (0.1)	0/429 (0.0)	2/930 (0.2)	1/1185 (0.1)
		Possible	1375/3419 (40.2)	247/429 (57.6)	291/930 (31.3)	310/1185 (26.2)
		Unlikely	345/3419 (10.1)	97/429 (22.6)	61/930 (6.5)	83/1185 (7.0)

WHO-UMC: World Health Organisation-Uppsala Monitoring Center
